# Improving Knowledge that Alcohol Can Cause Cancer is Associated with Consumer Support for Alcohol Policies: Findings from a Real-World Alcohol Labelling Study

**DOI:** 10.3390/ijerph17020398

**Published:** 2020-01-07

**Authors:** Ashini Weerasinghe, Nour Schoueri-Mychasiw, Kate Vallance, Tim Stockwell, David Hammond, Jonathan McGavock, Thomas K. Greenfield, Catherine Paradis, Erin Hobin

**Affiliations:** 1 Public Health Ontario, Toronto, ON M5G 1V2, Canada; Ashini.Weerasinghe@oahpp.ca (A.W.); Nour.Schoueri-Mychasiw@oahpp.ca (N.S.-M.); 2 Canadian Institute for Substance Use Research, University of Victoria, Victoria, BC V8P 2Y2, Canada; vallance@uvic.ca (K.V.); timstock@uvic.ca (T.S.); 3 School of Public Health and Health Systems, University of Waterloo, Waterloo, ON N2L 3G1, Canada; david.hammond@uwaterloo.ca; 4 Children’s Hospital Research Institute of Manitoba, University of Manitoba, Winnipeg, MB R3E 3P4, Canada; JMcGavock@chrim.ca; 5 Alcohol Research Group, Public Health Institute, Emeryville, CA 94608, USA; tgreenfield@arg.org; 6 Canadian Centre for Substance Use and Addiction, Ottawa, ON K1P 5E7, Canada; CParadis@ccsa.ca

**Keywords:** cancer prevention, alcohol, alcohol policy, alcohol warning labels

## Abstract

Knowledge that alcohol can cause cancer is low in Canada. Alcohol labels are one strategy for communicating alcohol-related harms, including cancer. Extending existing research observing an association between knowledge of the alcohol–cancer link and support for alcohol policies, this study examined whether increases in individual-level knowledge that alcohol is a carcinogen following an alcohol labelling intervention are associated with support for alcohol polices. Cancer warning labels were applied to alcohol containers at the intervention site, and the comparison site did not apply cancer labels. Pre-post surveys were conducted among liquor store patrons at both sites before and two-and six-months after the intervention was stopped due to alcohol industry interference. Limiting the data to participants that completed surveys both before and two-months after the cancer label stopped, logistic regression was used to examine the association between increases in knowledge and support for policies. Support for pricing and availability policies was low overall; however, increases in individual-level knowledge of the alcohol-cancer link was associated with higher levels of support for pricing policies, specifically, setting a minimum unit price per standard drink of alcohol (OR = 1.86, 95% CI: 1.11–3.12). Improving knowledge that alcohol can cause cancer using labels may increase support for alcohol policies. International Registered Report Identifier (IRRID): RR2-10.2196/16320

## 1. Introduction

Alcohol consumption is a leading risk factor for burden of disease, contributing to an estimated 3 million deaths (5% of all deaths) and 133 million disability-adjusted life years (DALYs) (5% of all DALYs) globally in 2016 [1]. The ethanol in alcoholic beverages has been classified as a Group 1 carcinogen (the highest category of risk) since 1988, and recent evidence indicated that alcohol consumption can cause at least seven types of cancers [2,3,4]. In 2016, alcohol use accounted for approximately 4% of cancer deaths and cancers were the predominant source of total alcohol-attributable deaths in higher-income countries, particularly among those over age 50 [5]. The relationship between alcohol consumption and increased risk of certain cancers has now been extended beyond heavy alcohol consumption to include low and moderate levels of consumption, and to all types of alcohol including wine, beer, and spirits [5,6]. 

Despite the serious health harms caused by alcohol, 2 billion people currently consume alcohol regularly, and global consumption is expected to increase 17% by 2030 [7]. In Canada, alcohol is the most widely used psychoactive substance (excluding caffeine), with 78% of Canadian adults aged 15+ (approximately 22 million) consuming alcohol in the past year and an annual per capita consumption of 8.2 litres, more than double the global average [8,9,10]. The number of hospitalizations fully attributable to alcohol across Canada is higher than hospitalizations from heart attacks and the annual cost of alcohol ($14.6B) exceeds the costs from cannabis and all illicit substances combined and is similar to tobacco [11,12]. Consistent with international data, the public perception of alcohol in Canada is that it is less harmful than tobacco and other substances, and it is not understood to be a carcinogen, or is only considered dangerous at high levels of consumption [13,14,15,16,17]. A recent Canadian survey also found that 69% of participants indicated that they would reduce their alcohol consumption if they learned that alcohol increases cancer risk [13]. Alcohol policy in Canada has been largely neglected relative to emerging initiatives addressing tobacco control, responses to the opioid overdose crisis and vaping, and restrictions imposed on the new legal cannabis market [12]. Coordinated, comprehensive, and effective policies are needed to increase awareness of the risks of alcohol and reverse trends of high alcohol-related harms and costs.

Studies have indicated that alcohol control policies have a protective effect against alcohol use and related harm [18,19]. Indeed, stricter alcohol policy environments can gradually shift a population towards lighter alcohol use [18] and are also associated with lower secondhand effects of alcohol [20]. While a large robust evidence base consistently suggests that restricting the availability and marketing and regulating the price of alcohol are the most cost-effective and easy-to-implement measures for reducing population-level alcohol consumption and harms [21,22,23,24,25,26,27,28,29,30], these policies often endure strong opposition from sections of the public and hence also some policy-makers [31,32,33]. Since alcohol is perceived by many as a relatively benign substance, trying to limit or contain alcohol consumption, even when supported by good evidence, is often resisted by some in the public who see availability, price and convenience of purchase as a societal good [34]. Public approval of alcohol policies is critical for social change. Information-based interventions, such as media campaigns, may be promising approaches for increasing knowledge of the negative consequences of alcohol and changing public opinion on alcohol policies shown to reduce per capita consumption and harms [35,36,37,38]. It is likely that the widespread awareness of health risks associated with smoking contributed to the public support for restrictive tobacco control policies [39]. Therefore, greater investment in educating the public about the risks associated with alcohol may be needed to create a more supportive environment for enacting effective alcohol control policies.

Alcohol container labels are one potentially effective information-based strategy for increasing consumer knowledge about the negative consequences of alcohol and are recommended by the World Health Organization [40,41]. Labels influence behaviour by attracting consumers’ attention and keeping the message in consumers’ minds through repeated exposure at key points of contact—the point-of-purchase and the point-of-pour [42]. Labels are appealing because of their low cost to regulators and unparalleled reach among drinkers, with the heaviest drinkers exposed most often [43]. Several lab and online studies testing alcohol warning messages have suggested that cancer warning labels are effective for increasing knowledge of alcohol as a risk factor for cancer and enhancing motivations to reduce alcohol use compared to other health messages [17,44,45,46,47].

**Figure 1:**
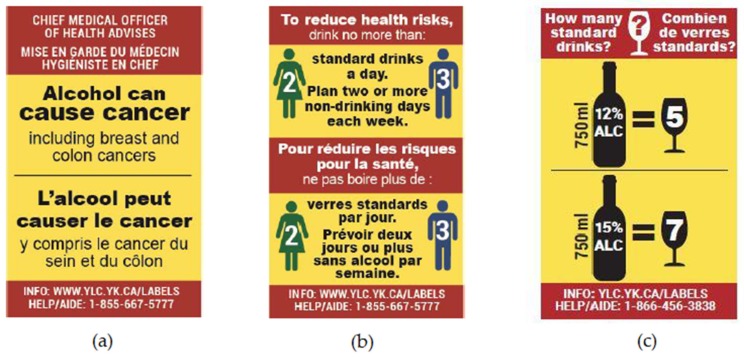
Intervention alcohol warning labels (actual size 5.0 cm × 3.2 cm): (**a**) Label 1: Cancer Warning; (**b**) Label 2: Canada’s Low-Risk Drinking Guidelines; (**c**) Label 3: Standard Drink Information (example for wine). Note: Alcohol containers sold in the liquor store in the intervention site were labelled with one of the three labels displayed in Figure 1.

A real-world alcohol labelling intervention study was implemented in Yukon, Canada with the purpose of testing the impact of the labels on consumer awareness of alcohol-related health harms, such as cancer and drinking behaviours. As shown in Figure 1, the alcohol labelling intervention consisted of three rotating labels: (1) a cancer warning including specific references to breast and colon cancers—two prevalent, often fatal cancers in Canada [48], (2) Canada’s low-risk drinking guidelines, and (3) standard drink information (four separate labels were developed for wine, spirits, coolers, and beer). Consistent with previous studies examining effective product label design, the intervention labels were relatively large in size to make them easily noticed and read, were full colour with a bright yellow background and red border so that they stand out on the container, and had messages providing new information and were rotated to avoid wear out [41,47,49,50,51,52,53,54,55]. A recent evaluation of the label intervention conducted by Hobin et al., on which the analyses of this paper are also based, found that participant knowledge of the link between alcohol and cancer was approximately 25% before the alcohol labelling intervention [56]. These findings are consistent with the low levels of awareness of the alcohol-cancer link in national estimates in Canada in 2008, and are also similar to findings from surveys in Australia and the UK [14,57,58]. Extending existing cross-sectional research from Europe and Australia observing an association between awareness of alcohol as a carcinogen and support for alcohol control policies [35,36,37,38], the main objective of this paper was to examine whether increases in individual-level knowledge of the link between alcohol and cancer following an alcohol labelling intervention are associated with stronger support for alcohol policy measures.

## 2. Materials and Methods 

### 2.1. Study Design 

The study design has been described elsewhere [59]. Briefly, two waves of longitudinal surveys were scheduled among cohort participants in the intervention and comparison sites four months before (wave 1: May–June 2017) and eight-months after (wave 2: May–June 2018) the intervention labels were implemented. The two intervention labels with a cancer warning and national drinking guidelines were applied to all alcohol containers in the intervention site, with the exception of select local and single serve beer and cider (~3% of products), starting 20 November 2017. The standard drink labels were to be introduced shortly thereafter. However, one month into the eight-month study period, the government in the intervention site halted its participation in the study due to significant pressure from Canada’s alcohol producers and stopped applying labels [59,60]. Based on the remaining label stock, approximately 47,000 cancer warning labels and 53,000 national drinking guidelines labels were applied to alcohol containers within the one-month period. In April 2018, the government in the intervention site resumed their participation in the study under the condition that the cancer warning label be excluded from rotation. Thus, the drinking guidelines label and the standard drink label were reinstated from April to July 2018. The study design was modified due to the interruption in the intervention, and wave 2 surveys were conducted in February 2018 in order to capture the impact of the shortened intervention. Wave 3 surveys were conducted from June 2018 to the end of the study period in July 2018. In waves 2 and 3, participants who provided their contact information in a preceding wave were emailed survey instructions, a unique survey link, and an e-transfer as remuneration. Additionally, due to attrition, the sample was replenished using wave 1 recruitment procedures in liquor stores in both sites. All the survey periods lasted six weeks, the surveys took approximately 18 minutes to complete, and survey protocols and measures were consistent across waves and sites. The survey was completed in English only as the majority of the residents in the intervention and comparison sites speak English at home and work [61,62]. The study was approved by the Research Ethics Boards at Public Health Ontario (ID 2017-010.04) and the University of Victoria (Protocol 17-161).

### 2.2. Participants

Participants were recruited by trained research assistants as they exited liquor stores in both the intervention and comparison sites using a standard intercept technique of approaching every person that passed a pre-identified landmark in the liquor store. At the time of initial recruitment, participants were adults of legal drinking age (19+), current drinkers (≥1 drink in past 30 days), residing in the intervention and comparison sites, bought alcohol at the liquor store, and did not report being pregnant or breastfeeding. 

### 2.3. Measures

#### 2.3.1. Knowledge of Alcohol as a Carcinogen

Knowledge of alcohol as a carcinogen was measured by asking participants, “Based on what you know or believe, can drinking alcohol cause…?” for each of breast cancer, liver disease, the flu, and [when pregnant cause] harm to unborn babies. Only responses to the cancer item are reported here. Responses for “breast cancer” were dichotomised as ‘Caused by alcohol’ (Yes) versus ‘Not caused by alcohol’ (No/Don’t know). Given that in the alcohol label intervention, the label with the cancer warning was implemented on alcohol containers in the liquor store in the intervention site between waves 1 and 2 only, “increases” in knowledge of alcohol as a carcinogen following the alcohol labelling intervention is defined in this paper as a participant who responded ‘Not caused by alcohol’ in wave 1 and responded ‘Caused by alcohol’ in wave 2 for breast cancer.

#### 2.3.2. Support for Alcohol Policies

**Figure 2:**
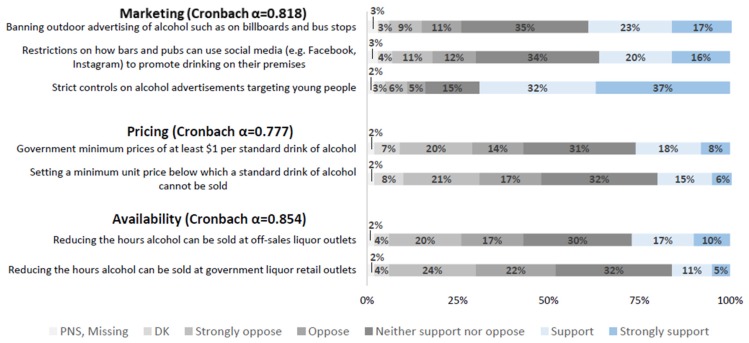
Level of support for alcohol policies from participants’ last survey wave completed. Percent missing varies from 1.16% to 1.56% for seven policy measures. Note: DK = Don’t know, PNS = Prefer not to say.

Support for alcohol policies was measured using seven items previously used in research conducted in Australia and the UK [35,36,37]. These items were first introduced in the wave 2 survey, with no wave 1 data available. To assess support for alcohol policies, participants were asked, “To reduce the problems associated with excessive alcohol use, to what extent would you support or oppose each of the following policies…?”, followed by a list of seven policy options targeting alcohol price, availability, and marketing (Figure 2). These policy options were adapted from the study by Buykx et al., [37] as well as a public opinion survey conducted by the Yukon government to inform the local liquor act in 2017 [63]. Responses were recorded on a five-point Likert scale (1 = Strongly oppose and 5 = Strongly support), and included ‘Don’t know’, ‘Prefer not say’ and ‘Missing’ as options.

#### 2.3.3. Socio-Demographics

Socio-demographic measures included age, sex, ethnicity (White, Aboriginal, and Other/Don’t know/Prefer not to say/Missing), education [low (completed high school or less), medium (completed trades or college certificate, some university or university certificate below Bachelor’s), high (university degree or post-graduation), and unknown (Don’t know/Prefer not to say/Missing)], and income [low (<$30,000), medium ($30,000–$59,999), high (≥$60,000), and unknown (Don’t know/Prefer not to say/Missing)]. 

#### 2.3.4. Other Covariates

Health literacy was assessed using the Newest Vital Sign assessment tool [64] and responses were categorized as limited (≤1 correct responses), possibility of limited (2–3 correct responses), adequate literacy (4–6 correct responses), and unknown (Don’t know/Prefer not to say/Missing). Alcohol use was measured using the quantity/frequency method [65]. Participants were asked to indicate how often they drank alcoholic beverages in the past six months and how many drinks they usually drank per occasion. Responses were combined to provide a mean number of drinks per week and categorized using Canada’s low-risk drinking guidelines: low risk (≤10 for females/15 for males per week), risky (11–19/16–29 per week), high risk (≥20/30 per week) [66], and unknown (Don’t know/Prefer not to say/Missing). 

### 2.4. Statistical Analysis

The analyses involved four stages. Firstly, to investigate trends in participant responses between policy measures within each of the three policy domains and determine if the policy measures could be combined, the reliability of internal consistency was measured using the Cronbach alpha coefficient (α). Values for α range from 0–1, with higher values indicating better reliability. Good reliability was established for all three alcohol policy domains (Cronbach α = 0.854 for availability, 0.777 for pricing, and 0.818 for marketing) and thus, the measures in each domain were combined into a single outcome for price, availability, and marketing. Secondly, responses for support within each policy domain was dichotomised into “Support” (either Strongly support or Support any of the policy measures within each domain) or “Do Not Support” (either Strongly oppose, Oppose, neither support nor oppose, or Don’t know to all of the policy measures within each domain). Thirdly, to estimate predictors of policy support, three separate logistic regression models were conducted with the three policy domains, price, availability, and marketing, as outcomes. Socio-demographics, alcohol use and knowledge that alcohol can cause cancer were entered as independent variables. Ethnicity was defined as White vs. Other (Aboriginal/Other/Don’t know/Prefer not to say/Missing). Education, income, and health literacy were found to be correlated; thus, to improve the stability of the models, only education was used. Policy support measures were first introduced in the wave 2 survey; thus, data from survey waves 2 and 3 only were used in these analyses, using responses from participants’ last wave completed. Lastly, to examine whether increases in knowledge of the alcohol–cancer link following the alcohol labelling intervention are associated with support for alcohol policies, three logistic regression analyses were conducted using data limited to participants that completed surveys both in waves 1 and 2, adjusting for socio-demographics and alcohol use. A sensitivity analysis evaluated the effect of adjusting for heavy episodic drinking to improve estimation of alcohol consumption, as previous research suggested that the quantity/frequency method alone is a relatively weak measure of alcohol use [67]. This analysis did not substantially alter the main results in terms of direction, magnitude and statistical significance; thus, to increase the study power and efficiency, the results presented were not adjusted for heavy episodic drinking. As per agreement with the local territorial government partners, ethnicity is included in the sample description and adjusted for in the analyses, but not reported in the results. All analyses were performed using SAS 9.3 (SAS Institute Inc., Cary, NC, USA, 2013). 

## 3. Results

In total, 1730 unique participants were included in this study (Table 1). The overall response rate was 8.6% [68], with 53.2% of participants retained at wave 2 and 47.5% retained at wave 3. At the time of initial recruitment, 32.0% of participants were aware of the alcohol link: 23.0% from wave 1, 53.4% from wave 2, and 23.7% from wave 3. Participants lost to follow-up across the three survey waves were more likely to be younger, male, have lower education, income and literacy, consume high or unknown levels of alcohol, and be in the comparison site.

### 3.1. Associations Between Knowledge of Alcohol as a Carcinogen and Support for Alcohol Policies

In the participants’ last survey in either of waves 2 or 3, 37.0–37.2% were aware that alcohol can cause cancer, and the level of support for each of the seven alcohol pricing, availability, and marketing policies are presented in Figure 2. Knowledge of alcohol as a carcinogen was significantly associated with support for policies controlling alcohol pricing (OR = 1.87, 95% CI: 1.51–2.32), availability (OR = 1.62, 95% CI: 1.30–2.01), and marketing (OR = 1.44, 95% CI: 1.12–1.86) (Table 2). 

Support for alcohol pricing policies was associated with having a high level of education (OR = 1.59, 95% CI: 1.15–2.20) and not supporting alcohol pricing polices was associated with risky levels of alcohol use (OR = 0.55, 95% CI: 0.35–0.86), relative to the referent. Support for alcohol availability policies was associated with being in the intervention site (OR = 1.36, 95% CI: 1.08–1.72), female (OR = 1.31, 95% CI: 1.06–1.63), and aged 45+ (OR = 2.06, 95% CI: 1.27–3.33), relative to the referent. Similarly, support for alcohol marketing policies was associated with being female (OR = 1.51, 95% CI:1.20–1.92), aged 25–44 (OR = 1.80, 95% CI: 1.19–2.71) and 45 + (OR = 2.41, 95% CI: 1.60–3.63), and having a high level of education (OR = 1.84, 95% CI: 1.30–2.59), relative to the referent. Not supporting alcohol marketing policies was associated with unknown (OR = 0.55, 95% CI: 0.37–0.82) levels of alcohol use, relative to the referent. 

### 3.2. Associations between Increases in Knowledge of Alcohol as A Carcinogen and Alcohol Policies

Among the participants that completed surveys in both waves 1 and 2, an increase in knowledge of alcohol as a carcinogen was observed among 20.2–20.3% of participants. An increase in knowledge that alcohol is a carcinogen, relative to those without an increase, was positively associated with support for policies controlling alcohol pricing (39.1% vs. 26.5%; OR = 1.86, 95% CI: 1.11–3.12). While a higher proportion of those with an increase in knowledge that alcohol can cause cancer, relative to those without an increase in knowledge, supported policies controlling alcohol availability (27.3% vs. 25.2%; OR = 1.15, 95% CI: 0.66–1.99) and marketing (81.6% vs. 75.8%; OR = 1.40, 95% CI: 0.73–2.71), these differences did not reach conventional levels of statistical significance (Table 3).

## 4. Discussion

Knowledge that alcohol is a risk factor for cancer was associated with greater support for alcohol policies controlling price, marketing, and availability. These findings are in line with previous research examining associations between awareness of the alcohol–cancer link and policy support [35,36,37,38]. This study extended previous research by also examining the extent to which policy support is associated with increases in individual-level knowledge that alcohol can cause cancer among a prospective cohort of Canadian drinkers after an alcohol labelling intervention. Participants who became aware that alcohol can cause cancer between waves 1 and 2, before and after a one-month alcohol labelling intervention with a cancer warning label, were almost two times more likely to support alcohol pricing policies relative to individuals with no change in knowledge (i.e., individuals that were not aware that alcohol can cause cancer in wave 2 or that were already aware that alcohol can cause cancer in wave 1). Previous cross-sectional studies in Denmark and England have observed that TV and social media campaigns highlighting the link between alcohol and cancer were associated with higher levels of public awareness and support for alcohol policies [35,38]. Together, these findings suggest that consumer awareness of the health risks of alcohol can be increased and that increasing awareness may have an impact on public support for strengthening alcohol pricing policies, which if implemented, may increase health and wellness within society. 

Increasing individual-level knowledge of alcohol as a carcinogen was associated with higher levels of support for alcohol pricing policies in this study, specifically setting a minimum unit price per standard drink of alcohol. Research consistently indicates the positive impact of minimum unit pricing policies for alcohol control, particularly for reducing the sale and consumption of high-strength low-cost products known to cause the most alcohol-related harms [29,69]. A challenge, however, is gaining public support for such an initiative. The current study was conducted among a sample of drinkers (excluded abstainers) in two jurisdictions with the highest per capita alcohol consumption and costs due to alcohol-related harms in Canada [11]. Levels of support for alcohol pricing and availability policies were very low overall, with more participants being opposed to than supporting restrictions on pricing and availability policies. However, increasing individual-level knowledge of alcohol as a carcinogen was associated with higher levels of support for alcohol pricing policies in this study, specifically, setting a minimum unit price per standard drink of alcohol. One potential explanation for the association between increases in knowledge and support for minimum unit pricing policies in this study is that the negative consequences of alcohol are prevalent in the two study site communities [55] and the need for effective interventions at the population-level is urgent. Yet, alcohol pricing policies are relatively weak in these two jurisdictions compared to the rest of Canada. The alcohol pricing structures in Yukon and Northwest Territories do not currently include a set minimum unit price per standard drink of alcohol in off-premise retail outlets [70,71], and high strength alcoholic beverages can be sold at large discounted prices. Finnish and US studies have shown that support for health-related policies varies according to the experiences of survey participants, with higher levels of support for alcohol policies among those who have witnessed alcohol-related disturbances in their communities [72,73,74]. Alternatively, a Canadian study using aggregated data across the 10 provinces, but excluding the territories, found that higher-consuming jurisdictions are more likely to be biased towards opposing alcohol taxation policies [75]. Other possible explanations for the association between increased knowledge and support for alcohol pricing policy are that support for alcohol pricing policies was low to begin with in this study, particularly compared to support for marketing policies, and it might be easier to increase support levels of policy measures that are lower. Additionally, approximately 60% of the sample in the current study reported an annual income of at least $60,000, and high income populations are less sensitive to price interventions compared to low income populations [25,69]. It is not clear why increased knowledge of the alcohol–cancer link did not have a stronger association with support for alcohol availability policies in this study, with the exception that the policy measures used in this study examined support for reduced trading hours and the government-owned off-site retail outlets in the two study sites already limit trading hours to six days per week, liquor stores are closed on Sundays.

Largely consistent with previous cross-sectional research, this study found that knowledge of alcohol as a carcinogen was significantly associated with support for policies controlling alcohol availability, pricing and marketing. The magnitude of the associations were also consistent with previously published cross-sectional studies examining population-level effects and the association between increases in individual-level knowledge of the alcohol-cancer link and support for pricing policies observed in this study [36,37,38]. Other receiver characters associated with support for alcohol pricing, marketing, and availability policies in this study, such as being female, being of older age, having higher levels of education, and consuming lower levels of alcohol, are consistent with previous studies [35,37,76,77,78,79]. Moreover, this study also observed, regardless of knowledge of the alcohol–cancer relationship, higher levels of support for alcohol marketing policies, relative to policies controlling the hours alcohol can be sold in off-premise retail outlets and the minimum unit price of alcohol, which are largely consistent with research from the US, Europe, and Australia [35,36,37,38,76]. Support towards health behaviour policies that are perceived as less restrictive and target other people and not themselves tends to be higher, specifically policies intended to protect young people [80]. More research is needed to confirm the extent to which efforts to improve awareness that alcohol can cause cancer may reduce the knowledge deficit and positively influence support for these more restrictive policies among the public.

The main strength of our study is the use of prospective research in the context of an alcohol labelling intervention to examine whether increases in individual-level knowledge of alcohol as a carcinogen is associated with policy support. However, our study has limitations. First, this study used only one measure, specifically regarding breast cancer, to test respondents’ knowledge of alcohol as a carcinogen. Previous research examining knowledge of alcohol-related cancers report the lowest levels of knowledge for breast cancer compared to other cancers, such as liver and colon [14,17,81]. Future research could examine the association between knowledge of alcohol as a risk factor for specific types of cancers and support for alcohol policies. Next, a prompted measure of knowledge of alcohol as a carcinogen was used in this study. There is inconsistency in the literature as to whether prompted or unprompted cancer awareness is a stronger predictor of support for alcohol pricing, marketing, and availability policies [36,38]; thus, both prompted and unprompted cancer awareness measures should be used in future studies to predict support for alcohol policies. Additionally, support for alcohol policies was only measured in survey waves 2 and 3, and not measured in wave 1 in this study. Consequently, participants that only responded in wave 1 were excluded from analyses and prevented analyses examining relationships between changes in both knowledge of alcohol as a carcinogen and policy support. The odds ratios estimating an association between an increase in knowledge and support for alcohol availability and marketing policies did not reach levels of conventional statistical significance. The limited sample size may, at least in part, contribute to not reaching conventional levels of statistical significance, and producing relatively wide confidence intervals and less precise estimates. Lastly, the study cannot provide representative estimates of the population as the response rate was low and the participants were recruited from liquor stores in city centres using systematic recruitment methods. However, given the stores from which the customers were recruited are virtual monopolies for the off-premise sale of alcohol in both experimental sites, they will have been broadly representative of persons purchasing alcohol in those cities. Nevertheless, the sample in this study includes a large percentage of participants who self-reported low levels of alcohol consumption, a population shown to be less defensive to health warning messages relative to higher level consumers [82].

## 5. Conclusions

In the context of a short alcohol labelling intervention, support for alcohol policies affecting alcohol price, availability, and marketing was associated with knowledge that alcohol can cause cancer. Increases in individual-level knowledge that alcohol is a risk factor for cancer was also associated with greater support for alcohol pricing policies. Improving knowledge of alcohol consumption health risks, specifically cancer risk, using alcohol warning labels may be an effective strategy for increasing public support for effective alcohol control policies that are currently not well supported, and which, in return, may improve population health.

## Figures and Tables

**Table 1:** Sample characteristics by Knowledge of Alcohol-Cancer Link at time of initial recruitment (n = 1730).

Knowledge of Alcohol as a Carcinogen		
Characteristic	Not Caused by Alcohol (N = 1177)	Caused by Alcohol (N = 553)
	N (%)	N (%)
Wave of initial recruitment ***		
1	390 (33.14)	127 (22.97)
2	516 (43.84)	295 (53.35)
3	271 (23.02)	131 (23.69)
Site *		
Intervention	697 (59.22)	359 (64.92)
Comparison	480 (40.78)	194 (35.08)
Age [Mean (SD)]	44.82 (14.32)	44.65 (14.53)
**Age Categories**		
19–24	92 (7.82)	45 (8.14)
25–44	480 (40.78)	228 (41.23)
45+	605 (51.40)	280 (50.63)
**Ethnicity**		
White	799 (67.88)	386 (69.80)
Aboriginal	225 (19.12)	104 (18.81)
Other	153 (13.00)	63 (11.39)
Sex **		
Male	622 (52.85)	255 (46.11)
Female	555 (47.15)	298 (53.89)
Education Levels ***		
Low (Completed high school or less)	241 (20.48)	88 (15.91)
Medium (Trades or college certificate, some university or university certificate below Bachelor)	420 (35.68)	171 (30.92)
High (Bachelor degree or higher)	445 (37.81)	266 (48.10)
Unknown (DK, PNS, Missing)	71 (6.03)	28 (5.06)
**Income Levels**		
Low (<$30,000)	138 (11.72)	76 (13.74)
Medium ($30,000 to <$60,000)	188 (15.97)	85 (15.37)
High (≥$60,000)	713 (60.58)	341 (61.66)
Unknown (DK, PNS, Missing)	138 (11.72)	51 (9.22)
Alcohol Use *		
Low risk volume ≤ 10 for females/15 for males per week	866 (73.58)	402 (72.69)
Risky volume 11–19/16–29 per week	74 (6.29)	44 (7.96)
High risk volume ≥ 20/30 per week	114 (9.69)	70 (12.66)
Unknown (DK, PNS, Missing)	123 (10.45)	37 (6.69)
Health Literacy Levels **		
Limited literacy (score ≤ 1)	364 (30.93)	129 (23.33)
Possibility of limited literacy (score 2–3)	210 (17.84)	123 (22.24)
Adequate literacy (score 4–6)	527 (44.7)	266 (48.10)
Unknown (DK, PNS, Missing)	76 (6.46)	35 (6.33)

* Pearson X^2^ test, *p* < 0.05, ** Pearson *X*^2^ test, *p* < 0.01, *** Pearson *X*^2^ test, *p* < 0.001, DK = Don’t know, PNS = Prefer not to say.

**Table 2:** Adjusted odds ratios (OR) and 95% confidence intervals (CI) of support for policy by demographics, alcohol use and knowledge of alcohol as a carcinogen.

Variable			Availability (N = 1680)			Pricing (N = 1681)			Marketing (N = 1677)
	N	N (%)	Adjusted OR (95% CI) ^1^	N	N (%)	Adjusted OR (95% CI) ^1^	N	N (%)	Adjusted OR (95% CI) ^1^
**Knowledge of alcohol as a carcinogen**									
Not caused by alcohol	1055	278 (26.35)	1.00 (ref)	1056	277 (26.23)	1.00 (ref)	1056	767 (72.63)	1.00 (ref)
Caused by alcohol	625	232 (37.12)	1.62 (1.30, 2.01)	625	254 (40.64)	1.87 (1.51, 2.32)	621	499 (80.35)	1.44 (1.12, 1.99)
**Sex**									
Male	847	229 (27.04)	1.00 (ref)	847	254 (29.99)	1.00 (ref)	841	602 (71.58)	1.00 (ref)
Female	833	281 (33.73)	1.31 (1.06, 1.63)	834	277 (33.21)	1.09 (0.88, 1.35)	836	664 (79.43)	1.51 (1.20, 1.92)
**Age**									
19–24	128	24 (18.75)	1.00 (ref)	127	30 (23.62)	1.00 (ref)	127	72 (56.69)	1.00 (ref)
25–44	676	192 (28.40)	1.61 (0.99, 2.62)	675	223 (33.04)	1.43 (0.91, 2.25)	676	497 (73.52)	1.80 (1.19, 2.71)
45+	876	294 (33.56)	2.06 (1.27, 3.33)	879	278 (31.63)	1.38 (0.88, 2.17)	874	697 (79.75)	2.41 (1.60, 3.63)
**Education Level**									
Low	317	93 (29.34)	1.00 (ref)	319	83 (26.02)	1.00 (ref)	320	205 (64.06)	1.00 (ref)
Medium	585	151 (25.81)	0.80 (0.58, 1.10)	584	157 (26.88)	1.03 (0.74, 1.43)	583	424 (72.73)	1.15 (0.84,1.57)
High	705	244 (34.61)	0.94 (0.66, 1.34)	704	271 (38.49)	1.59 (1.15, 2.20)	704	593 (84.23)	1.84 (1.30, 2.59)
Unknown	73	22 (30.14)	0.96 (0.54, 1.72)	74	20 (27.03)	0.97 (0.54, 1.75)	70	44 (62.86)	1.11 (0.63, 1.96)
**Alcohol Use**									
Low Risk volume	1234	381 (30.88)	1.00 (ref)	1234	409 (33.14)	1.00 (ref)	1232	969 (78.65)	1.00 (ref)
Risky volume	126	34 (26.98)	0.82 (0.54, 1.26)	126	27 (21.43)	0.55 (0.35, 0.86)	125	95 (76.00)	0.97 (0.62,1.53)
High Risk volume	186	53 (28.49)	0.94 (0.66, 1.34)	186	58 (31.18)	0.97 (0.69, 1.38)	184	124 (67.39)	0.74 (0.52,1.06)
Unknown	134	42 (31.34)	1.11 (0.73, 1.69)	135	37 (27.41)	0.91 (0.60, 1.40)	136	78 (57.35)	0.55 (0.37, 0.82)
**Site**									
Comparison	643	162 (25.19)	1.00 (ref)	642	197 (30.69)	1.00 (ref)	641	451 (70.36)	1.00 (ref)
Intervention	1037	348 (33.56)	1.36 (1.08,1.72)	1039	334 (32.15)	1.02 (0.82,1.28)	1036	815 (78.67)	1.26 (0.99, 1.60)
**Wave**									
2	512	140 (27.34)	1.00 (ref)	517	155 (29.98)	1.00 (ref)	510	350 (68.63)	1.00 (ref)
3	1168	370 (31.68)	1.07 (0.84, 1.36)	1164	376 (32.30)	0.96 (0.75, 1.21)	1167	916 (78.49)	1.25 (0.97, 1.60)

^1^ Adjusted for knowledge of alcohol as a carcinogen, sex, age, ethnicity, education level, alcohol use, site, and wave. Note: Bold indicates *p* < 0.05.

**Table 3:** Adjusted odds ratios (OR) and 95% confidence intervals (CI) of support for policy by increase in knowledge of alcohol as a carcinogen.

Increase in Knowledge			Availability (N = 433 ^1^)			Pricing (N = 431 ^1^)			Marketing (N= 430 ^1^)
	N	N (%)	Adjusted OR (95% CI) ^2^	N	N (%)	Adjusted OR (95% CI) ^2^	N	N (%)	Adjusted OR (95% CI) ^2^
No	345	87 (25.22)	1.00 (ref)	344	91 (26.45)	1.00 (ref)	343	260 (75.80)	1.00 (ref)
Yes	88	24 (27.27)	1.15 (0.66, 1.99)	87	34 (39.08)	1.86 (1.11, 3.12)	87	71 (81.61)	1.40 (0.73, 2.71)

^1^ Includes participants that completed both survey waves 1 and 2. ^2^ Adjusted for increase in knowledge of alcohol as a carcinogen, sex, age, ethnicity, education level, alcohol use, and site. Note: Bold indicates *p* < 0.05.
